# Vision–language semantic guidance and topology refinement for robust retinal vessel segmentation and biomarker analysis in cerebral small vessel disease

**DOI:** 10.3389/fcell.2026.1940436

**Published:** 2026-09-23

**Authors:** Shanshan Hua, Tao Chen, Yuwei Mi, Yu Shen, Yisha Li, Jiong Zhang, Yunxin Ji

**Affiliations:** 1 The First Affiliated Hospital of Ningbo University, Ningbo, Zhejiang, China; 2 School of Biomedical Engineering, Nanjing University, Suzhou, China

**Keywords:** cerebral small vessel disease (CSVD), optical coherence tomography angiography, retinal microvasculature, retinal vascular biomarkers, segmentation, uncertainty-aware learning

## Abstract

**Background:**

Retinal microvascular alterations observed in optical coherence tomography angiography (OCTA) have emerged as promising biomarkers for cerebral small vessel disease (CSVD). However, accurate quantification of OCTA-derived vascular biomarkers remains challenging due to imaging noise, projection artifacts, low contrast, and structural discontinuities, which often lead to unreliable vessel segmentation and distorted vascular topology.

**Purpose:**

To address these challenges, we propose SemTopNet, a semantic‐topology joint modeling framework for OCTA vessel segmentation.

**Methods:**

The proposed framework integrates vision‐language semantic guidance, centerline-guided topology refinement, and uncertainty-aware adaptive supervision within a unified architecture. Specifically, semantic embeddings are incorporated to enhance global structural representation, while a centerline-guided topology refinement module explicitly improves vascular continuity and suppresses fragmented predictions. In addition, Monte Carlo dropout‐based uncertainty estimation is employed to improve robustness in ambiguous and low signal‐to‐noise regions.

**Results:**

Extensive experiments on a CSVD OCTA dataset demonstrate that SemTopNet consistently outperforms existing convolutional and Transformer‐based segmentation methods, achieving the best performance in Dice similarity coefficient (0.8931), clDice (0.9027), Recall (0.9293), and AUC (0.9567). Ablation studies further verify the effectiveness of semantic modeling, topology‐aware refinement, and uncertainty‐aware supervision. Based on the obtained segmentation results, quantitative retinal vascular biomarkers were further analyzed, revealing significant layer-dependent microvascular alterations in CSVD patients, particularly in the deep vascular complex (DVC).

**Conclusion:**

These findings demonstrate that topology-consistent OCTA vessel segmentation is essential for reliable retinal microvascular quantification. The proposed SemTopNet provides a robust framework for OCTA biomarker extraction and retinal‐cerebral vascular association analysis in CSVD‐related studies.

## 1 Introduction

Optical Coherence Tomography Angiography (OCTA) enables non-invasive and layer-resolved visualization of the retinal microvascular network, providing quantitative measurements such as vessel density, skeleton length, fractal dimension, and foveal avascular zone (FAZ) area (Yao et al., 2020). Given the developmental, anatomical, and physiological similarities between the retinal and cerebral microvasculature, increasing attention has been directed toward investigating OCTA-derived vascular metrics as potential imaging biomarkers for cerebral small vessel disease (CSVD) and related neurodegenerative disorders (Wang et al., 2018). Several studies have reported reduced retinal vessel density and capillary rarefaction in CSVD patients, with significant correlations to MRI markers such as white matter hyperintensity burden (Huang et al., 2024; Chen et al., 2025). These findings suggest that retinal microvascular alterations may reflect cerebral microvascular injury.

However, the reliability of OCTA-based structural biomarkers critically depends on accurate vessel segmentation. Unlike conventional fundus photography, OCTA images are inherently affected by speckle noise, projection artifacts, motion distortions, and signal attenuation, all of which may disrupt vascular continuity and introduce false flow signal. Prior studies (Li Z. et al., 2024; Holmen et al., 2020) have highlighted these imaging artifacts and segmentation inaccuracies as persistent methodological challenges that limit the robustness and reproducibility of OCTA-based quantitative analysis.

Existing deep learning–based OCTA vessel segmentation methods are predominantly pixel-driven convolutional models. Early approaches, represented by U-Net–style encoder–decoder architectures, focused on improving pixel-wise overlap accuracy through multi-scale feature fusion and attention mechanisms. (Gao et al., 2025) proposed a lightweight full-resolution CNN that extends the U-Net backbone with multi-scale feature extraction and attention mechanisms. DeepVasNet (Raja et al., 2024) incorporated a U-Net with a pretrained EfficientNet encoder and attention blocks to capture local vessel patterns. (Ye et al., 2022) proposed a multiscale feature interaction network with pyramid squeeze-and-excitation (SE) and coarse-to-fine modules to enhance vessel detail representation and handle variable vessel width.

While these models achieve competitive Dice scores, they primarily rely on local appearance cues and do not explicitly enforce structural connectivity. To address vessel discontinuity, topology-aware losses and skeleton-preserving constraints have been introduced. (Shit et al., 2021) proposed the clDice loss, which incorporates soft skeletonization into training to optimize centerline overlap and improve connectivity preservation. Deep Closing (Wu et al., 2024) learns shape priors and incorporates topology-focused refinement to enhance connected components in tubular structures. IterNet (Li et al., 2020) leverages structural redundancy within vascular networks through iterative refinement, improving the recovery of thin vessels. More explicitly, topology-aware approaches introduce connectivity-preserving constraints into the optimization objective. Despite their success in tubular structure segmentation, such topology-aware formulations remain underexplored in OCTA vessel segmentation. In addition, structural coherence remains sensitive to noise and local ambiguities inherent in OCTA imaging. Uncertainty estimation has further been explored to quantify prediction reliability under noise and distribution shifts. Bayesian formulations such as Monte Carlo dropout have been used to capture both aleatoric and epistemic uncertainty in segmentation networks, as first formalized by (Kendall and Gal, 2017). In retinal vessel segmentation, (Dang et al., 2025) introduced SAUNA, an uncertainty-aware framework that uses soft labels to improve retinal vessel segmentation, especially for thin and ambiguous vessels. These studies suggest that uncertainty maps could help highlight regions with low signal-to-noise or projection artifacts that might otherwise bias structural metrics, but joint modeling of uncertainty and vascular topology in OCTA segmentation remains an open research direction.

To facilitate reliable investigations of the association between retinal microvascular alterations and cerebral small vessel disease (CSVD), accurate extraction of OCTA-derived vascular structures is essential. However, structural biomarkers such as vessel density, skeleton length, and connectivity metrics are highly sensitive to segmentation errors, particularly discontinuities, spurious branches, and topology distortion. To address this challenge, we design Semantic–Topology Network (SemTopNet), a dedicated OCTA vessel segmentation framework that jointly models high-level structural semantics and global vascular topology. By integrating semantic constraints with topology-aware learning and uncertainty estimation, SemTopNet enhances structural continuity, suppresses false connections, and improves robustness under complex imaging conditions. The resulting high-fidelity segmentation enables more reliable quantitative biomarker extraction for downstream retinal–cerebral vascular analysis.

The contributions of this work are summarized as follows:

1. We propose SemTopNet, a unified OCTA vessel segmentation framework that jointly incorporates semantic modeling, topology preservation, and uncertainty estimation to enhance structural consistency and robustness.
2. We demonstrate that topology-consistent segmentation significantly improves the reliability of OCTA-derived vascular biomarkers, facilitating more accurate microvascular quantification.
3. We apply the proposed framework to retinal–cerebral association analysis and provide empirical evidence supporting the potential of OCTA biomarkers in CSVD-related disease studies.

## 2 Related work

### 2.1 Semantic modeling and vision–language

In recent years, vision–language pretraining models have introduced a new paradigm for semantic modeling in medical image analysis. Unlike conventional segmentation methods that rely solely on visual features, vision–language models establish cross-modal alignment between images and textual embeddings, enabling the encoding of higher-level semantic priors and structural knowledge, thereby enhancing global contextual understanding.

In general computer vision, CLIP demonstrated that large-scale image–text contrastive learning can achieve effective cross-modal embedding alignment, significantly improving generalization and semantic representation capabilities (Radford et al., 2021). In medical imaging, several studies have incorporated vision–language frameworks to inject clinical knowledge. For example, MedCLIP (Wang et al., 2022) and BioViL (Bannur et al., 2023) enhance visual representations by leveraging medical reports and self-supervised cross-modal learning. IFSeg further shows that semantic segmentation can be achieved without task-specific images by constructing artificial vision–language pairs based solely on category-level semantics (Yun et al., 2023).

Beyond pure vision–language frameworks, semantic-guided strategies have been widely explored to enhance feature representation and structural understanding. Representative works integrate semantic information into multimodal fusion and segmentation processes to improve structural consistency and feature discrimination (Zhou et al., 2025; Li J. et al., 2024; Wen et al., 2024). More recently, semantic guidance has been combined with foundation models and advanced architectures, such as language-prompt-driven segmentation and semantic graph reasoning, to further improve performance in complex scenarios (Li et al., 2026; Song et al., 2024).

Nevertheless, existing vision–language and semantic-guided approaches primarily focus on lesion detection, organ-level segmentation, or general structural enhancement. For fine, topology-sensitive microvascular networks, particularly in OCTA imaging, systematic exploration of how semantic priors can improve structural continuity and global topological consistency remains limited.

### 2.2 Topology and structural modeling in segmentation

Topology modeling in vascular segmentation primarily focuses on explicit structural constraints. clDice incorporates a differentiable soft-skeletonization operator into the training process, introducing centerline overlap into the loss function to enhance connectivity preservation while optimizing pixel-wise overlap metrics (Shit et al., 2021). Subsequent works further extend topology modeling by exploring connectivity-aware interaction mechanisms and topology contrastive constraints, aiming to enhance structural discrimination and consistency (Wang et al., 2024).

Beyond local skeleton consistency, topology-constrained approaches based on Persistent Homology directly optimize topological invariants, such as Betti numbers, to impose global structural consistency (Clough et al., 2020). Meanwhile, alternative strategies incorporate topology priors through generative modeling or graph-based representations, enabling implicit topology refinement and global structure reasoning (Araújo et al., 2019; Yao et al., 2023).

In retinal vessel segmentation, methods such as IterNet and recent full-resolution architectures further improve structural continuity by leveraging iterative refinement and multi-scale feature fusion (Li et al., 2020; Su et al., 2024).

Overall, existing topology-aware methods mainly emphasize structural regularization through loss functions or architectural design. In OCTA imaging, where noise, projection artifacts, and low signal-to-noise ratios are prevalent, how to jointly model structural constraints with high-level semantic guidance and prediction reliability remains an open problem.

### 2.3 Uncertainty modeling in segmentation

Medical image segmentation is often affected by imaging noise, annotation ambiguity, and distribution shift. Therefore, uncertainty modeling has been widely adopted to estimate predictive confidence and improve model reliability. Monte Carlo dropout, grounded in Bayesian inference, enables the joint modeling of epistemic (model) and aleatoric (data) uncertainty. This framework was systematically introduced by Kendall and Gal and has become a foundation for many subsequent uncertainty-aware approaches (Kendall and Gal, 2017).

For specific medical image segmentation tasks, Wang et al. proposed an aleatoric uncertainty estimation strategy based on Test-Time Augmentation (TTA), where multiple spatial perturbations of the input are used to approximate predictive distributions and mitigate overconfident mis-segmentations (Wang et al., 2019). Another line of research evaluated various uncertainty estimation methods, including Monte Carlo dropout, model ensembles, and deep ensembles, across multiple datasets and architectures. These studies demonstrated that uncertainty estimation can effectively identify unreliable predictions and facilitate segmentation quality control (Ng et al., 2022).

Uncertainty modeling has also been applied to vascular and organ segmentation tasks. For example, (Martin et al., 2019) explored the use of epistemic uncertainty to detect low-confidence regions in cardiovascular image segmentation, thereby assisting expert review and quality assurance. More recent Bayesian deep learning approaches further integrate multiple uncertainty metrics, such as predictive entropy and mutual information, with segmentation quality prediction to assess reliability at a global level (OK et al., 2025). In retinal vessel segmentation, Dang et al. proposed the SAUNA framework, which models annotation uncertainty via soft-label regression, leading to improved performance in thin vessels and boundary regions (Dang et al., 2025). Despite these advances, existing uncertainty-aware methods mainly focus on confidence estimation and error detection, and are rarely combined with structural connectivity or topology-consistency constraints.

## 3 Methods

### 3.1 Overall framework

As illustrated in Figure 1, we propose a semantic–topology joint modeling framework for OCTA vessel segmentation, termed the Semantic–Topology Network (SemTopNet), which integrates language-driven semantic constraints and global topological perception within a unified architecture.

**FIGURE 1:**
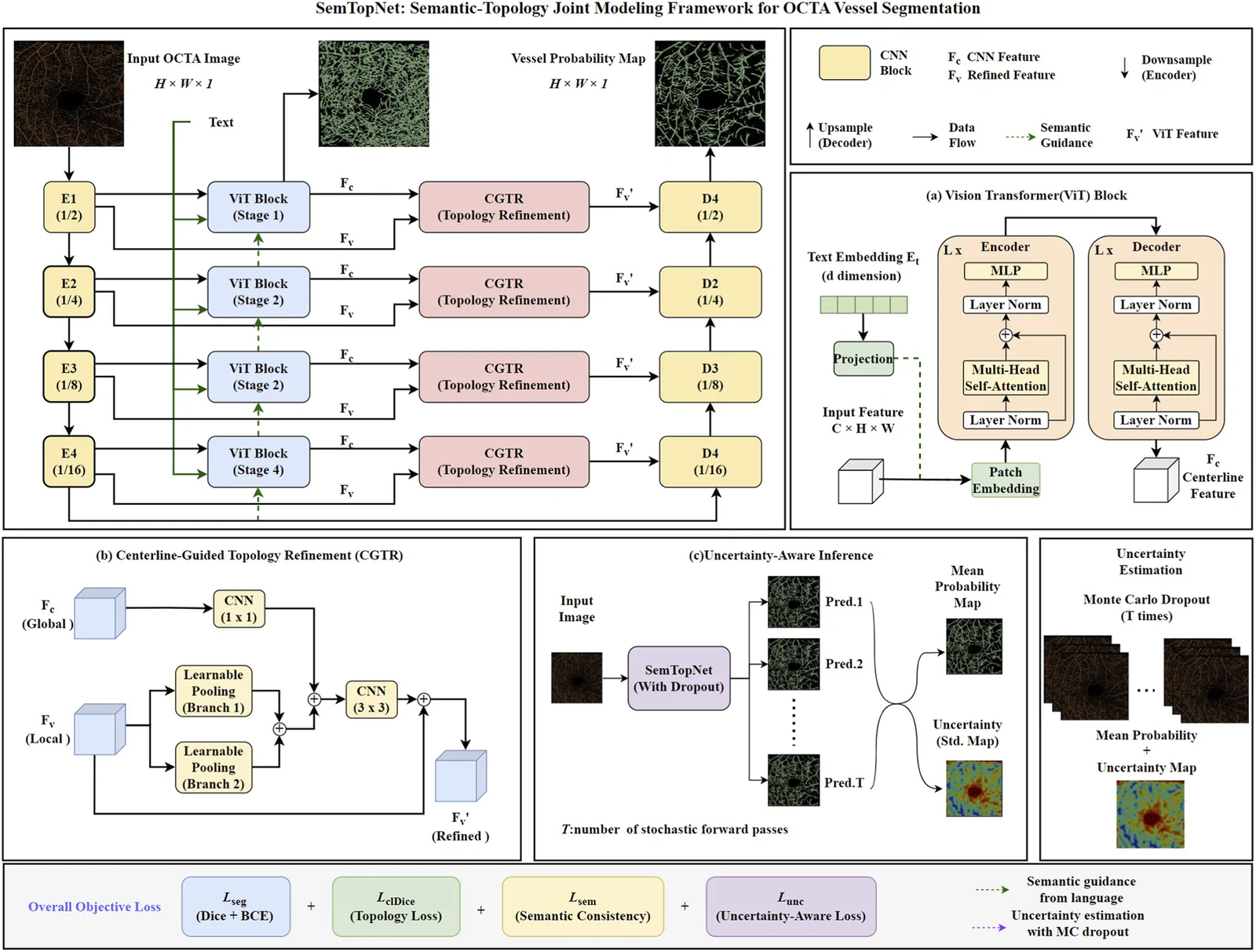
Overview of the proposed semantic–topology joint modeling framework for OCTA vessel segmentation. The framework integrates language-driven semantic constraints with centerline-guided topological continuity modeling.

Specifically, structural semantic representations are learned through cross-modal interaction between visual features and textual embeddings, enabling the network to encode high-level anatomical priors beyond purely appearance-based cues.

To further enhance vascular continuity and structural coherence, a centerline-guided continuity enhancement mechanism is incorporated to explicitly model global connectivity patterns. By jointly optimizing semantic consistency and topological regularity, SemTopNet achieves collaborative modeling of the overall vascular topology and fine-grained local details.

Under complex imaging conditions, including low contrast and capillary discontinuity, the proposed method significantly improves structural consistency, segmentation stability, and topological integrity of retinal microvasculature.

### 3.2 Vision–language semantic and topological joint modeling framework

To simultaneously characterize the global vascular topology and fine-grained local details, we construct a dual U-shaped encoder–decoder architecture. The overall framework consists of a Transformer-based semantic–topology branch and a convolutional segmentation branch, which are integrated through a centerline-guided enhancement module for multi-scale feature interaction.

In the semantic–topology branch, the input image is first projected into visual tokens via a convolutional embedding layer. To enhance high-level structural representation, semantic information describing retinal vascular characteristics is incorporated into the feature interaction process. These semantic representations are aligned with the visual features through attention-based interaction, allowing the network to emphasize vessel continuity, branching patterns, and global structural organization. The fused representations are then fed into a Vision Transformer (ViT) encoder to perform global modeling, capturing long-range spatial dependencies and the overall connectivity patterns of retinal vessels.

During the decoding stage, multi-scale representations are progressively recovered through hierarchical upsampling. This branch outputs a centerline feature map $F_{c}$ , which encodes the structural skeleton and global topological trend of the vascular network.

In parallel, a convolutional U-Net branch performs multi-scale feature extraction and reconstruction on the input image to generate vessel feature representations $F_{v}$ . This branch focuses on modeling local texture patterns and boundary information, ensuring pixel-level segmentation accuracy and detailed structural delineation.

To explicitly guide segmentation features with structural priors, a centerline enhancement module is introduced at corresponding resolution levels. Taking the centerline feature $F_{c}$ as a structural prior, the module conditionally modulates the vessel feature $F_{v}$ and compensates for connectivity discontinuities caused by weak local responses. Through cross-branch multi-scale interaction, the proposed architecture achieves collaborative modeling of semantic–topological information and fine-grained local features.

### 3.3 Centerline-guided topology refinement module (CGTR)

Due to the inherently low signal-to-noise ratio and locally weak responses in OCTA images, vessel segmentation results are prone to structural discontinuities, particularly in thin branches and low-contrast regions. To explicitly enhance vascular continuity and topological consistency, we propose a Centerline-Guided Topology Refinement (CGTR) module, which performs discrepancy-aware residual modulation between vessel features and centerline features.

Let the vessel feature map and centerline feature map be denoted as $F_{v}$ and $F_{c}$ , respectively. To suppress single-pixel noise and capture neighborhood structural trends, both features are first projected via a shared local convolutional operator (Equation 1):

$$P_{v}=G\left(F_{v}\right),\;P_{c}=G\left(F_{c}\right),$$ (1)

where G(⋅) represents a learnable local convolutional mapping.

To identify and compensate discontinuity-prone regions, we formulate a unified discrepancy-aware residual modulation (Equation 2):

$$F_{v}^{\prime }=F_{v}⊙\left(1+\sigma \;\left(\phi \;\left(ReLU\left(P_{c}-P_{v}\right)\right)\right)\right),$$ (2)

where ϕ(⋅) denotes a convolutional transformation and σ(⋅) is the Sigmoid activation function. This formulation highlights spatial locations where structural priors persist but vessel activation is insufficient, and adaptively amplifies weak responses while preserving stable regions.

To further strengthen global topological trunk information, we introduce a controlled fusion with centerline features (Equation 3):

$$F_{\text{out}}=F_{v}^{\prime }+\sigma \left(\psi \left(F_{c}\right)\right)⊙F_{c},$$ (3)

where ψ(⋅) is a learnable convolutional mapping. Through the adaptive fusion coefficient, the model incorporates structural priors without overwhelming local texture information, thereby improving overall topological integrity while avoiding excessive reliance on skeleton cues.

### 3.4 Uncertainty-aware adaptive supervision

In low signal-to-noise or structurally ambiguous regions, vessel predictions are often unstable and sensitive to feature perturbations. To improve robustness and suppress unreliable responses, we introduce an uncertainty-aware adaptive supervision strategy based on Monte Carlo Dropout.

During training, dropout layers remain activated and T stochastic forward passes are performed to obtain prediction samples ${\left\{P^{\left(t\right)}\right\}}_{t=1}^{T}$ . The predictive mean and variance are computed as Equation 4:

$$\bar{P}=\frac{1}{T}\overset{T}{\underset{t=1}{\sum }}P^{\left(t\right)},\;U=\frac{1}{T}\overset{T}{\underset{t=1}{\sum }}{\left(P^{\left(t\right)}-\bar{P}\right)}^{2}.$$ (4)

The variance map U reflects the spatial distribution of predictive uncertainty. Based on U , we construct a normalized pixel-wise weight map W and formulate an uncertainty-weighted cross-entropy loss (Equation 5):

$$L_{\text{unc}}=-\frac{\sum W\left[Y\log\left(\bar{P}+\epsilon \right)+\left(1-Y\right)\log\left(1-\bar{P}+\epsilon \right)\right]}{\sum W}.$$ (5)

This adaptive weighting mechanism aligns supervision strength with prediction stability, suppressing unreliable activations in uncertain regions while reinforcing confident structural responses.

### 3.5 Loss function

The training objective integrates segmentation accuracy, topological consistency, and uncertainty-aware supervision. Given the predicted vessel probability map P and the ground-truth annotation Y , we employ the Dice loss to alleviate class imbalance and enhance segmentation performance in thin-vessel regions (Equation 6):

$$L_{\text{seg}}=L_{\text{Dice}}\left(P,Y\right)+L_{\text{BCE}}\left(P,Y\right).$$ (6)

To explicitly enforce structural continuity, we further incorporate the clDice loss, which measures the agreement between the predicted segmentation and the skeletonized ground truth (Equation 7):

$$L_{\text{cl}}=\text{clDiceLoss}\left(P,Y\right)$$ (7)

The centerline branch is not supervised independently; its feature learning is implicitly optimized through the topology constraint imposed by $L_{\text{cl}}$ , serving as an intermediate structural representation.

To improve robustness in low signal-to-noise or structurally ambiguous regions, we adopt an uncertainty-aware adaptive supervision loss $L_{\text{unc}}$ , which weights the contribution of each pixel based on predictive uncertainty.

Combining these components, the overall objective function is defined as Equation 8:

$$L=L_{\text{seg}}+\lambda_{1}L_{\text{cl}}+\lambda_{2}L_{\text{unc}},$$ (8)

where $\lambda_{1}$ and $\lambda_{2}$ are balancing coefficients that control the relative contributions of topological consistency and uncertainty-aware supervision.

## 4 Experiment

### 4.1 Dataset

This study used OCTA images from patients with cerebral small vessel disease (CSVD) and healthy controls (HCs). The dataset was used for two different purposes: segmentation model training and downstream statistical biomarker analysis.

To train and evaluate a robust vessel segmentation model, the segmentation dataset was designed to capture diverse retinal vascular morphology and imaging conditions across both diseased and healthy eyes. Therefore, OCTA images from 120 CSVD patients and 40 healthy controls were used. Because this was an image-level segmentation task rather than an eye-level clinical comparison, eligible OCTA images from both eyes of the same participant were allowed to be included. Disease labels were not used as segmentation targets; instead, the model was trained to segment retinal vessels from OCTA images using manual vessel annotations as ground truth.

All OCTA data were acquired using a Heidelberg OCTA system (Heidelberg Engineering, Germany) with a scanning area of 3 mm × 3 mm centered on the macular region. The acquired data were processed into two-dimensional en-face projections with a resolution of 512×512 pixels. En-face images from the superficial vascular complex (SVC), intermediate vascular complex (IVC), and deep vascular complex (DVC) were exported for subsequent analysis. Images with insufficient signal strength, severe motion artifacts, projection artifacts, or other obvious image-quality defects were excluded before segmentation and quantitative analysis.

Vessel annotations were manually delineated by two experienced ophthalmologists and served as the ground truth for segmentation experiments. In addition, ground-truth skeletons were generated by skeletonizing the manual vessel annotations. These skeletons were used only for computing the topology-aware clDice loss and clDice-based evaluation, rather than being provided as additional network inputs or independent supervision targets for the centerline branch. To evaluate model generalization, the segmentation dataset was split at the participant level into training, validation, and test sets with a ratio of 60%, 20%, and 20%, respectively, ensuring that images from the same participant, including bilateral eye images when available, did not appear across different subsets.

For downstream statistical biomarker analysis, a balanced 1:1 cohort was constructed to ensure comparability between groups and reduce the potential influence of group-size imbalance. Therefore, 40 CSVD patients were selected from the larger CSVD cohort and compared with 40 healthy controls. To avoid within-subject correlation from bilateral eyes, the analysis was performed at the participant level, with only one eye included for each participant. When OCTA images from both eyes were available, the eye with better image quality, defined by higher signal strength and fewer motion or projection artifacts, was selected. When only one eye was available, that eye was included.

The study protocol was registered at Chictr.org.cn under the number ChiCTR2500098161 and was approved by the Ethics Committee of the First Affiliated Hospital of Ningbo University (No. 2023-103A). All participants provided written informed consent, and the study was conducted in accordance with the Declaration of Helsinki.

### 4.2 Implementation details

All models were implemented in PyTorch and trained on an NVIDIA GeForce RTX 3090 GPU. Model optimization was performed using the AdamW optimizer with an initial learning rate of $1\times 10^{-4}$ , a weight decay of $3\times 10^{-5}$ , and a batch size of 4. The network was trained for a maximum of 4,000 epochs. Validation was performed every five epochs, and the model achieving the best performance on the validation set was selected for final testing. A linear warm-up cosine annealing learning-rate schedule was adopted, with the first 400 epochs used for learning-rate warm-up. Batch normalization was employed within the network to stabilize feature distributions during training.

To improve model robustness and alleviate overfitting, data augmentation included random flipping, rotation, intensity scaling, and intensity shifting. The probabilities for random flipping and rotation were both set to 0.2, while the probabilities for random intensity scaling and intensity shifting were both set to 0.1. For uncertainty estimation, Monte Carlo dropout was performed with a dropout rate of 0.1, and (T = 8) stochastic forward passes were used to estimate the predictive mean and uncertainty. In the overall objective function, the weighting coefficients for the topology-aware clDice loss and uncertainty-aware loss were set to $\lambda_{1}=0.1$ and $\lambda_{2}=0.2$ , respectively. For inference, the predicted probability maps were converted into binary vessel masks using a threshold of 0.5.

### 4.3 Evaluation metrics

Segmentation performance was comprehensively evaluated using four complementary quantitative metrics, each reflecting different aspects of structural accuracy and robustness.

The Dice Similarity Coefficient (DSC) measures the overall overlap between the predicted segmentation $\hat{Y}$ and the ground truth Y (Equation 9):

$$\text{DSC}=\frac{2|Y\cap \hat{Y}|}{\left|Y|+|\hat{Y}\right|}.$$ (9)

DSC evaluates global region-level consistency and is widely used to assess segmentation accuracy.

To explicitly evaluate vascular connectivity and structural continuity (Equation 10), we adopt the centerline Dice (clDice), which measures overlap at the skeleton level (Equation 11):

$$T_{\text{prec}}=\frac{\left|S\left(\hat{Y}\right)\cap Y\right|}{\left|S\left(\hat{Y}\right)\right|},\;T_{\text{sens}}=\frac{\left|S\left(Y\right)\cap \hat{Y}\right|}{\left|S\left(Y\right)\right|},$$ (10)

$$\text{clDice}=\frac{2T_{\text{prec}}T_{\text{sens}}}{T_{\text{prec}}+T_{\text{sens}}}.$$ (11)

where S(⋅) denotes the skeletonization operation applied to the vessel mask. Unlike conventional overlap metrics, clDice is particularly sensitive to vessel discontinuities and topology disruption, making it suitable for evaluating topology-consistent segmentation.

Recall is employed to quantify the sensitivity of detecting vessel pixels (Equation 12):

$$\text{Recall}=\frac{TP}{TP+FN},$$ (12)

where TP and FN denote the numbers of true positive and false negative pixels, respectively. This metric reflects the model’s sensitivity to vessel pixels, which is particularly relevant for assessing missed detections in fine microvascular structures.

The Area Under the Receiver Operating Characteristic Curve (AUC) evaluates the model’s discriminative capability across varying decision thresholds (Equation 13):

$$\text{AUC}=\int_{0}^{1}TPR\left(FPR\right)\;dFPR,$$ (13)

where TPR and FPR denote the true positive rate and false positive rate, respectively. AUC provides a threshold-independent assessment of pixel-wise discriminative capability.

In addition to segmentation accuracy, vascular morphological biomarkers, including vessel density and vessel tortuosity, were further computed for downstream retinal–cerebral association analysis in CSVD-related studies.

### 4.4 Comparative experiments

To comprehensively evaluate the effectiveness of the proposed method, six representative 2D retinal vessel segmentation approaches were selected for comparison, including MFI-Net (Ye et al., 2022), AMD-UNet (Yuan et al., 2022), OCT^2^-Former (Tan et al., 2023), IterNet (Li et al., 2020), U-Net (Ronneberger et al., 2015), nnUNet (Ronneberger et al., 2015), MTR-DNET (Chen et al., 2026) and ResUNet (Li et al., 2019). These methods cover classical convolutional architectures, multi-scale feature fusion networks, residual-enhanced variants, and Transformer-based global modeling approaches, enabling systematic comparison from diverse architectural perspectives.

Qualitative comparisons are presented in Figure 2. The first row focuses on vascular bifurcation regions to evaluate structural continuity. Although most competing methods are able to segment the main vessel trunks, discontinuities or local fragmentation frequently appear at branch points. Although ResUNet also achieves relatively good continuity, it fails to link the key positions closely enough, and there is also an overall excessive segmentation issue. Compared with ResUNet, our method has better ability to learn the shape of blood vessels. Our method produces more continuous and topologically consistent vessel structures, particularly at complex bifurcation regions. This improvement can be attributed to the centerline-guided constraint and the global structural modeling strategy, which enhance long-range dependency modeling and explicitly enforce vascular connectivity. The second row evaluates robustness under noisy or low-contrast conditions. Several comparison methods exhibit over-segmentation or spurious responses in noisy backgrounds, while others tend to miss weak capillary signals. Benefiting from the uncertainty-aware modeling mechanism, our approach effectively suppresses unreliable predictions and reduces noise-induced artifacts, leading to cleaner vessel maps and more stable segmentation performance.

**FIGURE 2:**
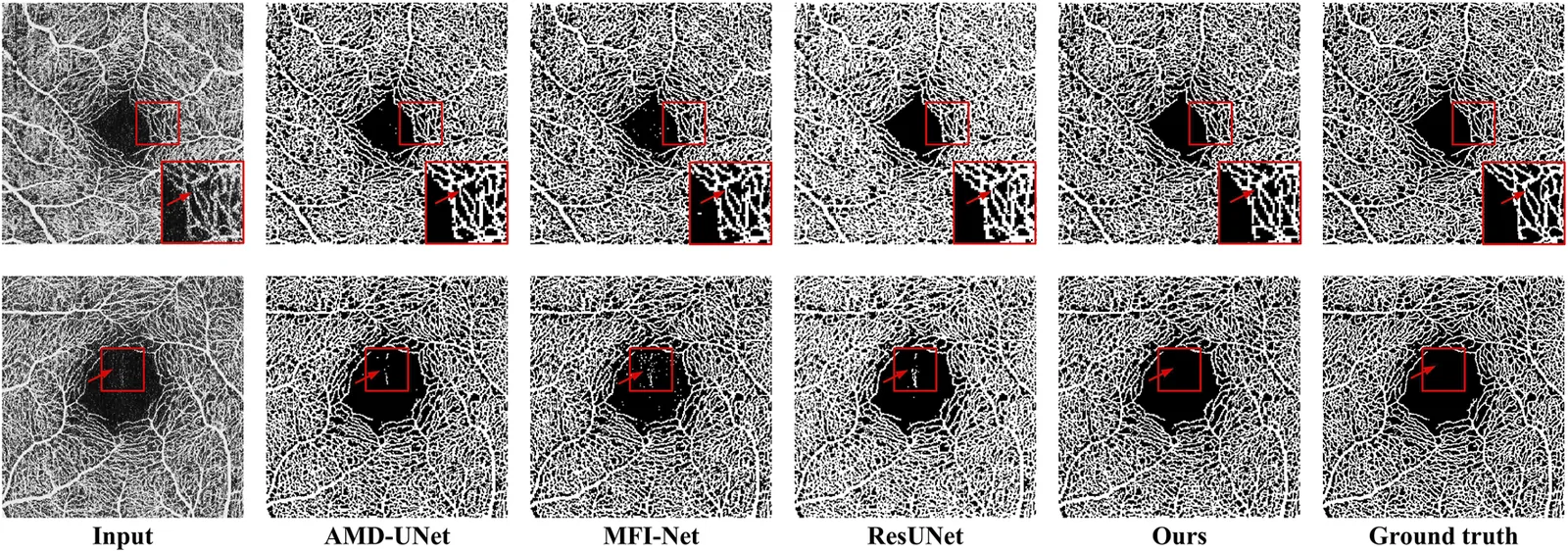
Qualitative comparison of different 2D retinal vessel segmentation methods. Two representative cases are randomly selected for visualization.

Overall, the proposed method not only preserves the continuity of major vessels but also better maintains fine vascular structures and structural integrity under complex anatomical configurations and challenging imaging conditions, including noise and low contrast.

Quantitative results are summarized in Table 1. Overall, the proposed SemTopNet achieves the best performance across all evaluation metrics, demonstrating consistent improvements over existing convolutional and Transformer-based approaches. Notably, SemTopNet attains the highest clDice score (0.9027), indicating superior topology preservation and enhanced structural continuity of the segmented vascular network. Since clDice explicitly measures skeleton-level overlap and connectivity, this improvement indicates the effectiveness of the proposed topology-aware modeling strategy in maintaining vascular integrity, particularly at bifurcation and thin-capillary regions. In addition, SemTopNet achieves the highest Recall (0.9293), suggesting improved sensitivity in detecting fine microvascular structures. This result reflects the benefit of joint semantic modeling and topology guidance, which together mitigate vessel fragmentation and reduce missed detections in low-contrast areas. The proposed framework also obtains the best DSC (0.8931) and AUC (0.9567), indicating improved overall segmentation accuracy and vessel–background discrimination capability. The higher AUC reflects a stronger threshold-independent ability to distinguish vessel pixels from background pixels. Together with the improvement in Recall, these results suggest that the uncertainty-aware strategy contributes to more stable vessel prediction under noisy and low-contrast conditions. The performance gain in AUC further suggests that uncertainty-aware estimation effectively suppresses unreliable responses under noisy conditions, improving prediction stability.

**TABLE 1:** Quantitative comparison of 2D retinal vessel segmentation methods (mean ± std).

Method	DSC	clDice	Recall	AUC
ResUNet (Li et al., 2019)	0.8656±0.0332	0.8970±0.0375	0.8737±0.0516	0.9201±0.0343
U-Net (Ronneberger et al., 2015)	0.8714±0.0576	0.8928±0.0623	0.8877±0.0852	0.9279±0.0336
nnUNet (Isensee et al., 2021)	0.8814±0.0429	0.8875±0.0465	0.9117±0.0554	0.9362±0.0418
MTR-DNET (Chen et al., 2026)	0.8887±0.0433	0.8903±0.0377	0.9025±0.0472	0.9219±0.0505
OCT^2^-Former (Tan et al., 2023)	0.8620±0.0307	0.8942±0.0331	0.9113±0.0348	0.9382±0.0308
MFI-Net (Ye et al., 2022)	0.8824±0.0373	0.8962±0.0385	0.9134±0.0322	0.9519±0.0390
IterNet (Li et al., 2020)	0.8616±0.0536	0.8921±0.0590	0.9073±0.0841	0.9538±0.0353
AMD-UNet (Yuan et al., 2022)	0.8850±0.0315	0.8949±0.0307	0.9019±0.0217	0.9093±0.0312
**SemTopNet (Ours)**	0.8931±0.0336	0.9027±0.0339	0.9293±0.0333	0.9567±0.0336

These results collectively demonstrate that jointly incorporating semantic modeling, topology preservation, and uncertainty estimation leads to structurally consistent and robust vessel segmentation. Such topology-consistent segmentation is essential for reliable OCTA-derived vascular biomarker quantification, thereby supporting downstream retinal–cerebral association analysis in CSVD-related studies.

### 4.5 Ablation study

To further investigate the contribution of each module, a systematic ablation study was conducted. U-Net was adopted as the baseline model (M0), upon which the semantic modeling module (+Sem, M1), the topology-guided centerline branch (+Cen, M2), and the uncertainty estimation mechanism (+UNC, M3) were progressively integrated.

Quantitative results are summarized in Table 2. Compared with the baseline U-Net, incorporating semantic modeling yields consistent improvements across all metrics, with DSC increasing from 0.8714 to 0.8862 and Recall improving from 0.8877 to 0.9061. The improvement in AUC (0.9279 → 0.9459) indicates enhanced vessel–background discrimination capability. These results demonstrate that high-level semantic priors and contextual feature fusion contribute to more complete vessel extraction and improved global representation.

**TABLE 2:** Ablation study of different modules in SemTopNet (mean ± std).

Model	DSC	clDice	Recall	AUC
M0: U-Net	0.8714 ± 0.0576	0.8928 ± 0.0623	0.8877 ± 0.0852	0.9279 ± 0.0336
M1: M0 + Sem	0.8862 ± 0.0329	0.8970 ± 0.0340	0.9061 ± 0.0240	0.9459 ± 0.0316
M2: M1 + Cen	0.8917 ± 0.0328	0.9018 ± 0.0338	0.9061 ± 0.0287	0.9525 ± 0.0311
M3: M2 + UNC	**0.8931** ± **0.0336**	**0.9027** ± **0.0339**	**0.9293** ± **0.0333**	**0.9567** ± **0.0336**

Further introducing topology guidance through the centerline branch leads to additional performance gains, particularly in clDice (0.8970–0.9018). Since clDice explicitly evaluates skeleton-level agreement, this improvement confirms that topology-aware supervision effectively enhances structural continuity and reduces branch disconnections. The simultaneous increase in DSC further indicates that structural constraints improve overall segmentation consistency without sacrificing regional accuracy.

Finally, integrating uncertainty estimation produces the full model and results in the most substantial Recall improvement (0.9061–0.9293), together with further gains in AUC (0.9525–0.9567). The pronounced increase in Recall suggests that uncertainty-aware supervision reduces false negatives and better preserves fine microvascular structures under noisy or low-contrast conditions. Meanwhile, the AUC improvement reflects enhanced robustness across varying decision thresholds.

Overall, the progressive improvements validate that semantic modeling enhances contextual representation, topology guidance enforces structural integrity, and uncertainty estimation improves predictive reliability. Their joint integration leads to structurally consistent and robust vessel segmentation, particularly beneficial for microvascular analysis in CSVD cases.

## 5 Discussion

### 5.1 Definition of quantitative parameters

Based on prior studies (Huang et al., 2024; Chen et al., 2025) linking retinal microvascular alterations to CSVD, a set of OCTA-derived quantitative parameters was selected to characterize foveal morphology, vascular density, and network topology. All parameters were computed separately in the superficial vascular complex (SVC), intermediate vascular complex (IVC), and deep vascular complex (DVC).

The foveal avascular zone (FAZ) was characterized using three geometric descriptors: FAZ area (FA), FAZ circularity (FC), and FAZ aspect ratio (FAR). FA measures the size of the FAZ region. FC, defined as $4\pi \cdot \text{Area}/{\text{Perimeter}}^{2}$ , quantifies the regularity of its shape. FAR represents the ratio of the major to minor axis of the fitted ellipse and reflects morphological elongation. These three parameters were calculated for the full FAZ region without subregional division.

Vascular density (Huang et al., 2024; Chen et al., 2025) was assessed using vessel area density (VAD) and vessel length density (VLD). VAD quantifies the proportion of perfused vasculature within the analyzed region, whereas VLD measures the total length of vessel centerlines normalized by area, providing a sensitive indicator of capillary loss.

Vascular morphology was further described using tortuosity, defined as the ratio of vessel path length to the corresponding chord length, averaged across all vessel segments.

To capture network complexity, fractal dimensio (FD) was computed using the box-counting method. In addition, the number of vessel branch points (BP) was used to quantify topological complexity, representing the total count of branching junctions in the vascular network.

Vascular parameters, including VAD, VLD, tortuosity, FD, and BP, were calculated independently for each retinal layer, including the SVC, IVC, and DVC. FAZ-related measurements, including FA, FC, and FAR, were calculated only in the IVC, because they are defined for the foveal avascular region rather than for each vascular plexus separately. Subregional analysis based on the Early Treatment Diabetic Retinopathy Study (ETDRS) grid (Early Treatment Diabetic Retinopathy Study Research Group, 1987) was performed for VAD, VLD, FD, tortuosity, and BP, as described in the following subsection. Layer-level biomarkers were computed over the entire en-face image of each vascular plexus and were used for global between-group comparisons. For ETDRS subregional analysis, the same biomarker categories were further quantified within predefined regional masks to characterize spatial heterogeneity. Therefore, Table 3 reports global layer-level measurements, whereas Table 4 reports sector-level regional measurements.

**TABLE 3:** Comparison of microvascular parameters between the CSVD and HC groups across different retinal layers.

Layer	Metric	HC (mean ± SD)	CSVD (mean ± SD)	p -value
SVC	VAD	0.4688±0.0231	0.4744±0.0285	0.105
	VLD	9615.04±403.81	9401.17±483.27	<0.001
	Tortuosity	8.29±7.25	7.76±6.41	0.403
	FD	1.923±0.012	1.920±0.015	0.037
	BP	5263.06±378.30	5035.63±448.56	<0.001
DVC	VAD	0.4909±0.0731	0.4628±0.0666	<0.001
	VLD	9510.30±1004.01	9263.03±875.32	<0.001
	Tortuosity	5.02±1.90	3.92±1.49	<0.001
	FD	1.929±0.016	1.914±0.017	<0.001
	BP	5238.55±897.70	4999.94±792.08	<0.001
IVC	FA	17.46±7.60	18.34±7.70	0.395
	FC	0.661±0.114	0.621±0.124	0.001
	FAR	1.162±0.126	1.192±0.157	0.086
	VAD	0.4932±0.0282	0.5008±0.0339	0.047
	VLD	9565.38±361.92	9353.06±418.20	<0.001
	Tortuosity	11.05±9.97	9.97±9.22	0.339
	FD	1.933±0.013	1.930±0.015	0.142
	BP	5176.00±337.01	4951.24±376.96	<0.001

**TABLE 4:** Statistical comparison results between CSVD and healthy controls across different ETDRS subregions.

Region	Metric	SVC			DVC			IVC		
​	​	HC (mean ± std)	CSVD (mean ± std)	p-value	HC (mean ± std)	CSVD (mean ± std)	p-value	HC (mean ± std)	CSVD (mean ± std)	p-value
SI	VAD	0.0346 ± 0.0032	0.0345 ± 0.0040	0.8207	0.0376 ± 0.0066	0.0346 ± 0.0065	<0.001	0.0369 ± 0.0037	0.0367 ± 0.0043	0.7361
	VLD	1.3976 ± 0.0988	1.3325 ± 0.1258	<0.001	1.3939 ± 0.1693	1.3027 ± 0.1714	<0.001	1.4080 ± 0.1007	1.3443 ± 0.1244	<0.001
	FD	1.6134 ± 0.0494	1.6070 ± 0.0586	0.3663	1.6333 ± 0.0426	1.6068 ± 0.0690	0.0002	1.6284 ± 0.0504	1.6205 ± 0.0550	0.2227
	Tortuosity	10.1212 ± 5.2082	8.6341 ± 4.5531	0.0092	10.0890 ± 4.7823	6.7867 ± 3.8695	<0.001	11.0504 ± 5.2350	9.5014 ± 4.8909	0.0047
	BP	426.5250 ± 55.1419	387.1127 ± 62.1979	<0.001	433.6026 ± 81.2588	381.6152 ± 78.1616	<0.001	430.7625 ± 54.7633	394.1294 ± 58.3104	<0.001
TI	VAD	0.0355 ± 0.0029	0.0355 ± 0.0034	0.8248	0.0368 ± 0.0053	0.0341 ± 0.0060	<0.001	0.0376 ± 0.0033	0.0375 ± 0.0039	0.9243
	VLD	1.4131 ± 0.0811	1.3565 ± 0.1034	<0.001	1.3951 ± 0.1352	1.3022 ± 0.1659	<0.001	1.4116 ± 0.0677	1.3596 ± 0.1045	<0.001
	FD	1.6253 ± 0.0428	1.6168 ± 0.0473	0.1555	1.6258 ± 0.0483	1.6038 ± 0.0709	0.0045	1.6354 ± 0.0442	1.6264 ± 0.0481	0.1527
	Tortuosity	5.6689 ± 2.4911	5.2796 ± 2.3043	0.1735	5.0669 ± 2.0867	4.2327 ± 2.0039	<0.001	6.2936 ± 2.4620	5.7172 ± 2.4297	0.0241
	BP	436.4500 ± 45.4159	402.3976 ± 58.7578	<0.001	429.4805 ± 72.8088	382.3916 ± 76.1065	<0.001	431.9125 ± 39.7021	401.9057 ± 55.7131	<0.001
II	VAD	0.0348 ± 0.0031	0.0348 ± 0.0034	0.9717	0.0373 ± 0.0062	0.0347 ± 0.0059	<0.001	0.0371 ± 0.0036	0.0370 ± 0.0041	0.8500
	VLD	1.3893 ± 0.0957	1.3376 ± 0.1152	0.0001	1.3895 ± 0.1620	1.3091 ± 0.1499	<0.001	1.3984 ± 0.0864	1.3497 ± 0.1198	<0.001
	FD	1.6203 ± 0.0447	1.6089 ± 0.0470	0.0456	1.6355 ± 0.0397	1.6071 ± 0.0479	<0.001	1.6301 ± 0.0440	1.6185 ± 0.0488	0.0444
	Tortuosity	9.1424 ± 4.4693	8.6078 ± 4.4656	0.1932	10.2896 ± 4.7785	6.9758 ± 3.9570	<0.001	9.9289 ± 4.7899	9.6714 ± 4.7076	0.5864
	BP	423.4250 ± 51.3183	390.7770 ± 62.3608	<0.001	427.5513 ± 72.3860	384.8309 ± 75.8790	<0.001	427.1125 ± 48.1382	398.1647 ± 58.8607	<0.001
NI	VAD	0.0356 ± 0.0047	0.0352 ± 0.0040	0.1154	0.0371 ± 0.0060	0.0342 ± 0.0056	<0.001	0.0373 ± 0.0047	0.0374 ± 0.0039	0.5571
	VLD	1.4041 ± 0.1386	1.3541 ± 0.1299	<0.001	1.4001 ± 0.1671	1.3170 ± 0.1464	<0.001	1.4089 ± 0.1224	1.3684 ± 0.1012	<0.001
	FD	1.6294 ± 0.0496	1.6144 ± 0.0626	0.0319	1.6339 ± 0.0480	1.6048 ± 0.0475	<0.001	1.6366 ± 0.0457	1.6276 ± 0.0486	0.1517
	Tortuosity	5.9744 ± 2.6270	5.5381 ± 2.3980	0.0506	5.9017 ± 2.4514	4.2333 ± 2.0122	<0.001	6.4928 ± 2.6145	6.0112 ± 2.4123	0.0300
	BP	433.0875 ± 66.8111	403.7559 ± 62.8483	<0.001	433.7692 ± 78.0912	389.4583 ± 75.0225	<0.001	435.5750 ± 58.2308	408.3412 ± 53.8126	<0.001
SE	VAD	0.0566 ± 0.0055	0.0567 ± 0.0085	0.2763	0.0605 ± 0.0087	0.0565 ± 0.0105	<0.001	0.0595 ± 0.0061	0.0598 ± 0.0093	0.1238
	VLD	1.9628 ± 0.2064	1.8861 ± 0.2789	0.0079	1.8899 ± 0.3559	1.8457 ± 0.3508	0.0871	1.9302 ± 0.2023	1.8456 ± 0.2763	0.0025
	FD	1.6855 ± 0.0265	1.6807 ± 0.0363	0.3842	1.6930 ± 0.0296	1.6773 ± 0.0466	0.0015	1.6931 ± 0.0264	1.6898 ± 0.0410	0.9876
	Tortuosity	11.0718 ± 5.2918	10.2734 ± 5.4730	0.1503	10.8197 ± 4.9482	9.9324 ± 5.0803	0.0916	10.9528 ± 5.0540	11.0144 ± 5.3202	0.9642
	BP	587.8375 ± 84.5113	552.8612 ± 106.1274	0.0052	557.2564 ± 148.1856	540.7980 ± 140.4204	0.1592	565.1750 ± 89.1301	527.2979 ± 101.6369	0.0014
TE	VAD	0.0559 ± 0.0078	0.0566 ± 0.0076	0.7605	0.0549 ± 0.0108	0.0532 ± 0.0087	0.0199	0.0585 ± 0.0083	0.0596 ± 0.0076	0.4594
	VLD	1.9747 ± 0.2136	1.9541 ± 0.2322	0.1935	1.9859 ± 0.3197	1.9593 ± 0.2698	0.0133	1.9440 ± 0.1991	1.9393 ± 0.2004	0.6965
	FD	1.6768 ± 0.0384	1.6727 ± 0.0437	0.2714	1.6719 ± 0.0423	1.6637 ± 0.0426	0.0273	1.6853 ± 0.0386	1.6823 ± 0.0390	0.2364
	Tortuosity	5.7586 ± 2.1907	5.3374 ± 2.2080	0.1111	5.4397 ± 2.2286	4.6874 ± 1.9692	0.0023	5.6033 ± 1.9921	5.6798 ± 2.2022	0.9770
	BP	593.0250 ± 81.3013	581.8635 ± 89.9814	0.2752	608.5065 ± 139.4108	593.7772 ± 115.7205	0.0166	570.3500 ± 78.7036	565.3349 ± 80.7526	0.6117
I.E	VAD	0.0556 ± 0.0060	0.0586 ± 0.0062	<0.001	0.0570 ± 0.0109	0.0565 ± 0.0093	0.3203	0.0591 ± 0.0064	0.0620 ± 0.0067	<0.001
	VLD	1.9816 ± 0.1901	1.9743 ± 0.1761	0.5149	1.9437 ± 0.3433	1.9508 ± 0.2805	0.2932	1.9556 ± 0.1952	1.9285 ± 0.1702	0.0636
	FD	1.6817 ± 0.0299	1.6808 ± 0.0307	0.8857	1.6757 ± 0.0644	1.6722 ± 0.0344	0.0397	1.6920 ± 0.0275	1.6902 ± 0.0312	0.9404
	Tortuosity	9.4306 ± 4.8739	10.0119 ± 4.8043	0.2388	10.6579 ± 5.5046	9.3018 ± 4.6973	0.0767	10.1792 ± 5.0287	11.0607 ± 4.9487	0.1329
	BP	600.5750 ± 79.8327	587.9601 ± 76.8899	0.1265	585.8205 ± 142.4299	584.1789 ± 122.4944	0.3592	577.4250 ± 85.1687	556.4282 ± 74.7105	0.0242
NE	VAD	0.0546 ± 0.0070	0.0533 ± 0.0104	0.9508	0.0523 ± 0.0088	0.0490 ± 0.0115	0.0068	0.0571 ± 0.0076	0.0559 ± 0.0117	0.8647
	VLD	1.9421 ± 0.1854	1.8616 ± 0.3463	0.2543	1.9509 ± 0.3037	1.8533 ± 0.3952	0.0367	1.9232 ± 0.1884	1.8450 ± 0.3587	0.5082
	FD	1.6765 ± 0.0289	1.6678 ± 0.0547	0.5957	1.6716 ± 0.0326	1.6515 ± 0.0690	0.0021	1.6844 ± 0.0274	1.6740 ± 0.0663	0.5630
	Tortuosity	5.1063 ± 2.1528	4.9248 ± 2.1323	0.5914	4.8250 ± 2.2042	4.4013 ± 1.9877	0.1250	5.5831 ± 2.2585	5.4217 ± 2.2398	0.6746
	BP	581.9367 ± 75.1384	553.4296 ± 119.5215	0.1379	604.4416 ± 125.3613	560.8627 ± 147.0491	0.0133	567.9241 ± 74.3365	541.6306 ± 120.0511	0.2880

### 5.2 Definition of subregions

To explore the spatial heterogeneity of retinal microvascular alterations in CSVD patients, we performed subregional analysis using a modified ETDRS macular grid. The center of the grid was defined as the foveal avascular zone (FAZ) centroid. The central circular area with radius R=0.5 mm was defined as the foveal region (not subdividing further). Surrounding this central area, an inner ring with outer radius 2R (i.e., 1 mm) and an outer ring with outer radius 3R (i.e., 1.5 mm) were defined. Each ring was divided into four quadrants (superior, temporal, inferior, nasal), yielding eight peripheral sectors: superior-internal (SI), temporal-internal (TI), inferior-internal (II), nasal-internal (NI), superior-external (SE), temporal-external (TE), inferior-external (I.E.,), and nasal-external (NE). The layout of these subregions and the retinal layer segmentation are illustrated in Figure 3.

**FIGURE 3:**
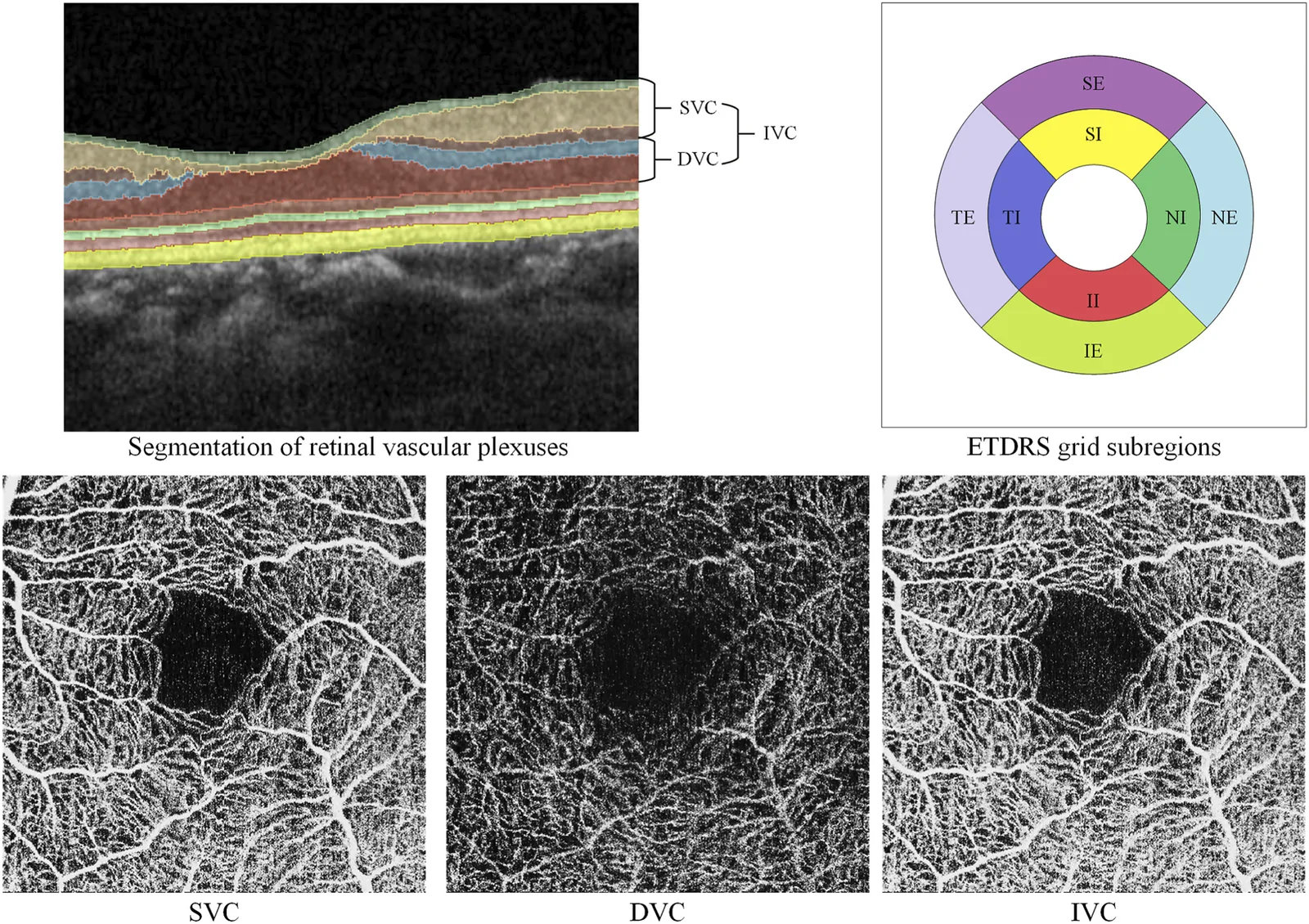
Illustration of retinal layer segmentation and ETDRS subregions.

The following parameters were computed within each of the eight peripheral sectors as well as within the central foveal region: vessel area density (VAD), vessel length density (VLD), fractal dimension (FD), tortuosity, and branch point count (BP). The FAZ geometric descriptors (FA, FC, FAR) were not subjected to subregional analysis, as they are defined only for the entire FAZ region.

### 5.3 Statistical analysis

Statistical analyses were performed to compare OCTA-derived microvascular parameters between the CSVD and HC groups. Continuous variables are presented as mean ± standard deviation. The normality of each parameter was assessed using the Shapiro–Wilk test. For normally distributed variables, between-group comparisons were performed using an independent-samples t -test. For non-normally distributed variables, the Mann–Whitney U test was used. All tests were two-sided, and statistical significance was defined as p<0.05 .

### 5.4 Quantitative analysis framework

In this study, retinal microvasculature was first segmented using the proposed method, yielding binary vessel maps that delineate the perfused vascular network. Based on the obtained vessel segmentation, the FAZ was subsequently estimated without requiring explicit FAZ annotations.

Specifically, the FAZ was approximated as the largest avascular region within the macular area. To this end, the vessel mask was inverted to obtain candidate non-perfused regions, followed by connected component analysis to identify contiguous avascular areas. The component corresponding to the FAZ was selected based on its spatial location and relative size, excluding the global background region. The centroid of the extracted FAZ region was then computed and used as an approximation of the foveal center for subsequent regional analysis.

In addition to the FAZ, the vascular skeleton was extracted to enable fine-grained quantification of vascular topology. The skeleton was obtained by applying morphological thinning to the binary vessel map, followed by pruning of small spurs. The resulting one-pixel-wide skeleton preserves the connectivity and branching structure of the retinal microvasculature, allowing the computation of vessel length density, tortuosity, and bifurcation points.

Figure 4 illustrates the intermediate results of the proposed pipeline. From left to right, the three panels show the binary vessel segmentation, the automatically detected FAZ mask, and the extracted vascular skeleton.

**FIGURE 4:**
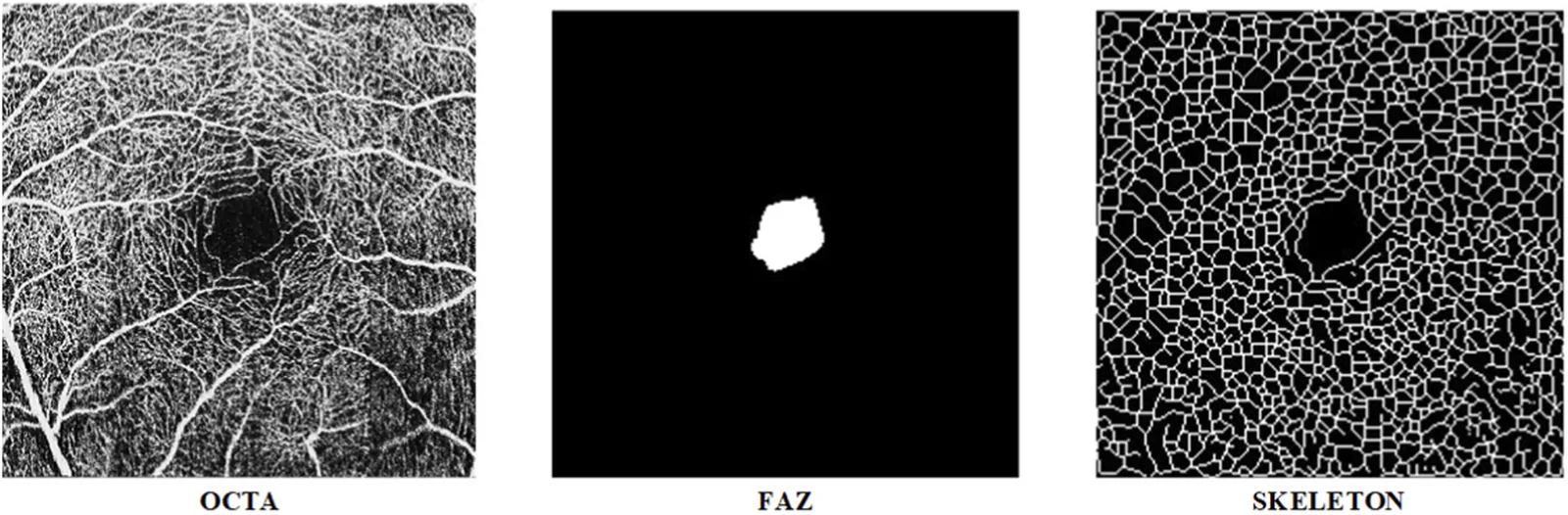
Key processing steps. Left: original vessel map. Middle: FAZ mask. Right: vascular skeleton.

### 5.5 Layer-dependent microvascular alterations in CSVD

As shown in Table 3 and Figure 5, the comparison of microvascular parameters between the CSVD and HC groups revealed distinct layer-dependent alterations. FAZ-related parameters, including FA, FC, and FAR, were evaluated only in the IVC because they are not anatomically defined in the SVC or DVC.

**FIGURE 5:**
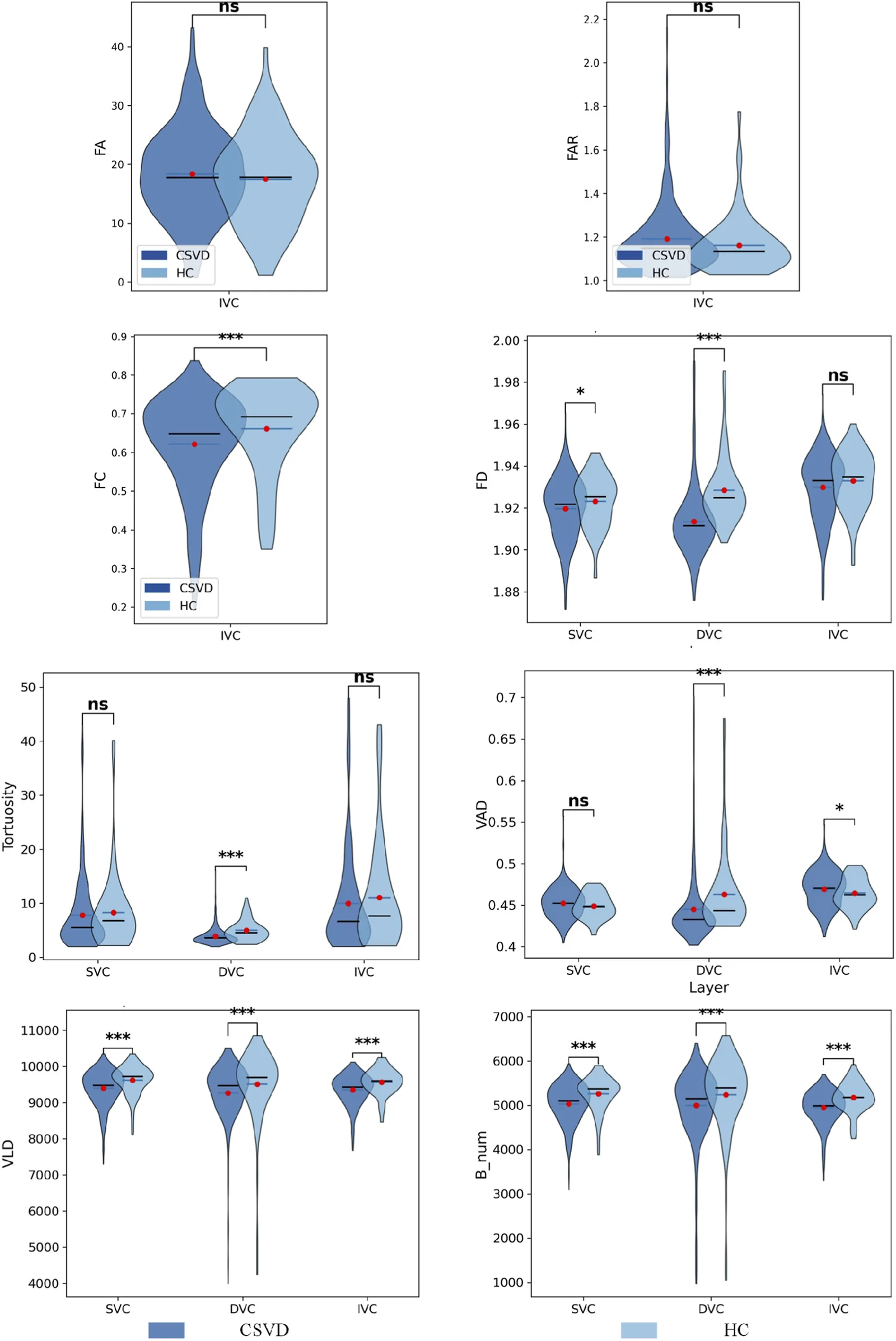
Distribution of microvascular parameters in the CSVD and HC groups across different retinal layers. Violin plots with overlaid boxplots show the distribution of each metric. Statistical significance between groups is indicated for each comparison.

In the SVC, three out of five parameters showed significant differences. Specifically, the CSVD group exhibited significantly lower VLD (p<0.001) , FD (p=0.037) , and BP (p<0.001) compared with HC. VAD did not differ significantly (p=0.105) , and tortuosity also showed no group difference (p=0.403) . These findings suggest that in the superficial plexus, vessel length density, fractal complexity, and branching-related metrics are more sensitive to CSVD-associated microvascular impairment than VAD or tortuosity.

In the DVC, the most extensive microvascular differences were observed. Compared with HC, the CSVD group showed significantly lower VAD, VLD, FD, BP, and tortuosity, with all parameters reaching statistical significance (p<0.001) . These results indicate that the deep vascular plexus may be particularly vulnerable to CSVD-associated microvascular alterations, involving reductions in vascular area, vessel length, branching complexity, and vessel tortuosity.

In the IVC, eight parameters were analyzed, including three FAZ-related metrics (FA, FC, and FAR). The CSVD group showed significantly lower FC (p=0.001) , VLD (p<0.001) , and BP (p<0.001) , whereas VAD was modestly but significantly higher in the CSVD group (p=0.047) . In contrast, FA (p=0.395) , FAR (p=0.086) , FD (p=0.142) , and tortuosity (p=0.339) did not reach statistical significance. Among the FAZ-related metrics, only FAZ circularity was significantly reduced in CSVD, whereas FAZ area and area ratio remained comparable between groups.

Several parameters showed consistent group differences across vascular layers. VLD and BP were significantly lower in the CSVD group in the SVC, DVC, and IVC, indicating widespread reductions in vessel length density and vascular branching complexity. FD was significantly lower in both the SVC and DVC but not in the IVC. In contrast, VAD showed layer-dependent changes, with a significant reduction in the DVC, a modest increase in the IVC, and no significant difference in the SVC. Tortuosity was significantly lower only in the DVC, with no differences in the SVC or IVC.

Importantly, FAZ-related parameters were exclusively assessed in the IVC, and only FC demonstrated a significant reduction in CSVD, suggesting that CSVD primarily affects the circularity rather than the overall size or relative area of the foveal avascular zone.

In summary, the DVC exhibited the greatest number and magnitude of significant microvascular alterations, followed by the IVC and then the SVC. These layer-dependent patterns may reflect differential susceptibility of the retinal capillary plexuses to cerebral small vessel disease. Unlike VLD and BP, which showed consistent reductions across all three vascular layers, VAD exhibited layer-specific changes, suggesting that area-based vascular measurements may be influenced by layer-specific vascular morphology or local redistribution of perfused vascular area.

### 5.6 ETDRS subregion analysis

Subregional analysis of the inner and outer ETDRS rings revealed spatially heterogeneous and layer-dependent microvascular alterations in the CSVD group, as illustrated in Figure 6 and summarized in Table 4. Overall, the inner ring showed more consistent group differences than the outer ring, and the DVC exhibited the most extensive alterations among the three vascular layers.

**FIGURE 6:**
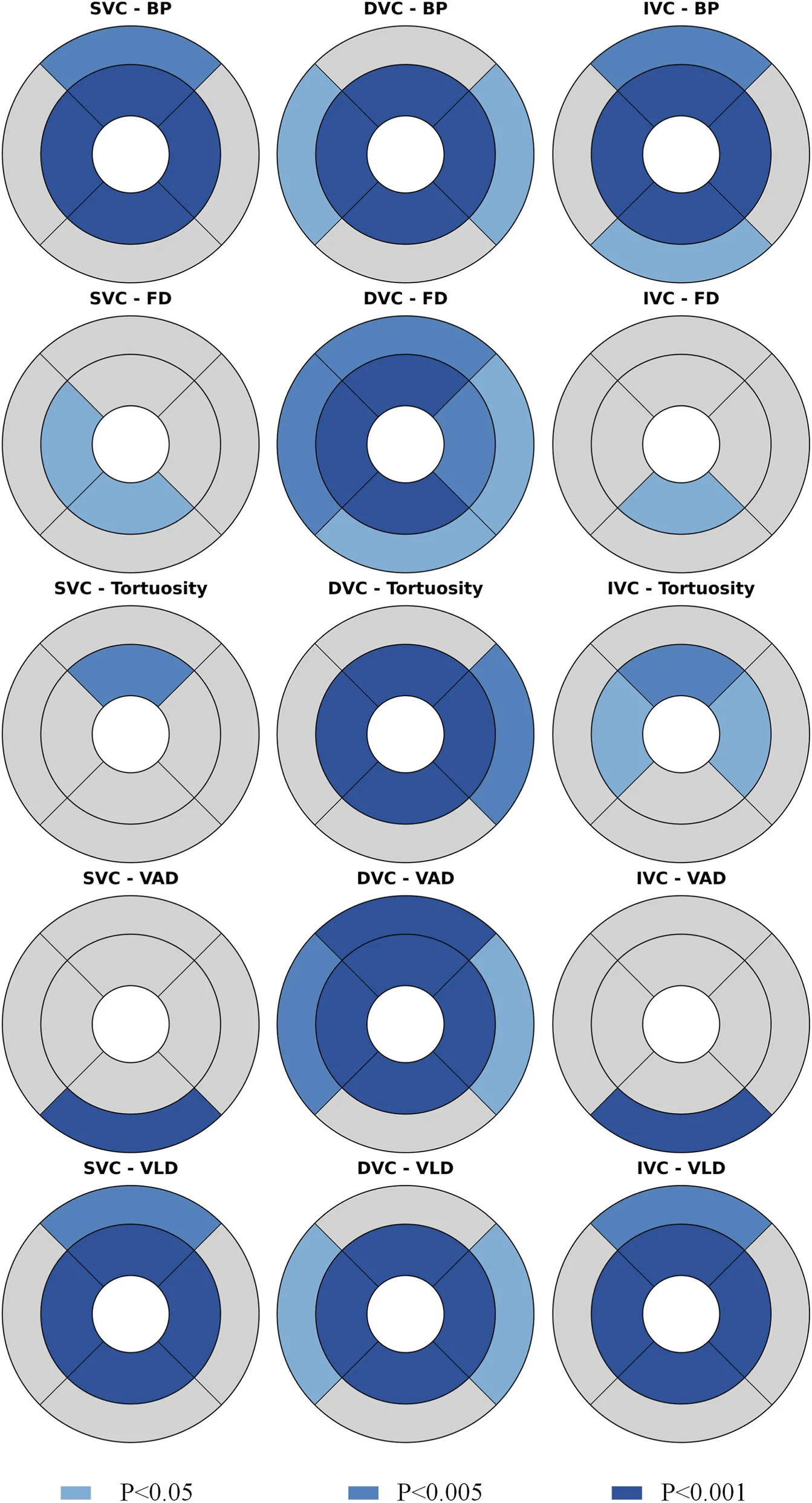
Spatial distribution of significant differences between CSVD and HC in ETDRS subregions.

In the SVC, significant differences were mainly observed in the inner ring. Within the four inner-ring quadrants, VLD and BP were significantly lower in the CSVD group than in HC (all p<0.001 ). Additional differences were observed for tortuosity in the superior-internal sector (p=0.009) and for FD in the inferior-internal (p=0.046) and nasal-internal (p=0.032) sectors. VAD did not show significant differences in any inner-ring quadrant. In the outer ring, the superior-external sector showed lower VLD (p=0.008) and BP (p=0.005) , whereas the inferior-external sector showed higher VAD in the CSVD group (p<0.001) . No significant differences were observed in the temporal-external or nasal-external sectors. These results suggest that SVC alterations in CSVD are primarily reflected by reductions in vessel length and branching complexity in the inner ring, with only limited outer-ring involvement.

The DVC showed the most extensive and consistent subregional alterations. In the inner ring, VAD, VLD, FD, tortuosity, and BP were significantly lower in the CSVD group across all four quadrants, with most comparisons reaching p<0.001 . In the outer ring, significant differences were also observed but were more spatially heterogeneous. The superior-external sector showed lower VAD (p<0.001) and FD (p=0.001) . The temporal-external sector showed lower VAD (p=0.020) , VLD (p=0.013) , FD (p=0.027) , tortuosity (p=0.002) , and BP (p=0.017) . The inferior-external sector showed lower FD (p=0.040) , whereas the nasal-external sector showed lower VAD (p=0.007) , VLD (p=0.037) , FD (p=0.002) , and BP (p=0.013) . These findings indicate that DVC involvement in CSVD is more diffuse than that in the SVC and IVC, with pronounced inner-ring impairment and selective extension into the outer ring.

In the IVC, the subregional pattern was intermediate between the SVC and DVC. In the inner ring, VLD and BP were significantly lower in the CSVD group across all four quadrants (all p<0.001 ). Tortuosity was also lower in the superior-internal (p=0.005) , temporal-internal (p=0.024) , and nasal-internal (p=0.030) sectors, while FD was lower only in the inferior-internal sector (p=0.044) . VAD did not differ significantly in the inner ring. In the outer ring, the superior-external sector showed lower VLD (p=0.002) and BP (p=0.001) , whereas the inferior-external sector showed higher VAD (p<0.001) and lower BP (p=0.024) . No significant differences were observed in the temporal-external or nasal-external sectors. These results suggest that IVC alterations are mainly characterized by reduced vessel length and branching complexity in the inner ring, with limited and region-specific changes in the outer ring.

Across the three vascular layers, VLD and BP were the most consistently altered subregional parameters, particularly in the inner ring. The DVC showed the broadest range of abnormalities, involving both density-related and topology-related metrics, whereas the SVC and IVC showed more restricted changes. Taken together, these findings suggest that CSVD-associated retinal microvascular alterations are spatially heterogeneous, with preferential involvement of the inner ETDRS ring and greater susceptibility of the deep vascular complex.

## 6 Conclusion

In this work, we proposed SemTopNet, a novel semantic–topology joint modeling framework for OCTA vessel segmentation, specifically designed to address the structural continuity and topology preservation challenges in microvascular imaging. By integrating vision–language driven semantic priors, a centerline-guided topology refinement module, and an uncertainty-aware adaptive supervision mechanism, SemTopNet achieves collaborative modeling of global vascular topology and fine-grained local details.

Extensive experiments on a CSVD OCTA dataset demonstrated that SemTopNet consistently outperforms existing convolutional and Transformer-based segmentation methods across all evaluation metrics, achieving the highest DSC (0.8931), clDice (0.9027), Recall (0.9293), and AUC (0.9567). Ablation studies further confirmed the individual contribution of each module, with uncertainty-aware supervision yielding the most substantial improvement in detecting fine microvascular structures.

Beyond segmentation accuracy, we applied SemTopNet to quantify layer-specific and spatially heterogeneous microvascular alterations in CSVD patients. Our results revealed that the deep vascular complex (DVC) is the most vulnerable layer, exhibiting widespread reductions in vessel density, fractal dimension, and branch points across both inner and outer ETDRS rings. The superficial and intermediate plexuses showed more restricted changes, primarily in the inner ring. Notably, FAZ circularity (FC) was significantly reduced in the CSVD group, while FAZ area and aspect ratio remained unchanged.

These findings demonstrate that topology-consistent segmentation is essential for reliable OCTA-derived biomarker extraction. The proposed SemTopNet framework provides a robust computational foundation for retinal–cerebral association studies and holds promise for broader applications in microvascular imaging tasks beyond CSVD.

## Data Availability

The original contributions presented in the study are included in the article/supplementary material, further inquiries can be directed to the corresponding authors.
